# Educational intervention on sexual satisfaction of Iranian men: application of the information, motivation and behavioral skills model

**DOI:** 10.3389/fpubh.2025.1533261

**Published:** 2025-05-15

**Authors:** Naseh Ghaderi, Fatemeh Zarei, Fazlollah Ghofranipour

**Affiliations:** ^1^Department of Health Education and Health Promotion, Faculty of Medical Sciences, Tarbiat Modares University, Tehran, Iran; ^2^Department of Health Education and Promotion, Boukan Faculty of Medical Sciences, Urmia University of Medical Sciences, Urmia, Boukan, Iran

**Keywords:** sexual satisfaction, marital relationship, sexuality education, Men’s health, IMB model

## Abstract

**Background:**

Marital satisfaction plays a critical role in fostering healthy relationships between couples, with sexual satisfaction being a key determinant. This study aimed to evaluate the impact of an educational intervention based on the Information–Motivation–Behavioral Skills (IMB) model on the sexual satisfaction of Iranian married men.

**Materials and methods:**

A quasi-experimental study was conducted among 100 married men in Sanandaj, Kurdistan, Iran. Participants were randomly assigned to either the intervention group, which received an online educational program, or the control group, which did not receive any structured education. The intervention utilized multimedia content, including videos, infographics, and interactive materials, to address three key domains: information, motivation, and behavioral skills related to sexual satisfaction. Data were collected through three validated instruments: the Sexual Health Questionnaire (based on the IMB model), the Male Sexual Function Scale, and Larson’s Sexual Satisfaction Scale. Assessments were conducted at baseline (pre-test) and 16 weeks after the intervention (post-test).

**Results:**

Significant improvements were observed in the intervention group compared to the control group across all (IMB) model constructs and sexual satisfaction measures (*p* < 0.05).

**Conclusion:**

The educational intervention based on the (IMB) model, delivered through an online multimedia platform, was effective in enhancing sexual satisfaction among the participants. This highlights the value of theory-driven, digitally delivered health education programs in improving men’s sexual health.

## 1 Introduction

Sex is a fundamental aspect of intimate relationships, playing a crucial role in shaping marital satisfaction between couples (1, 2). A fulfilling and positive sexual relationship has been consistently linked to enhanced well-being and overall life satisfaction in both partners (3). Sexual satisfaction, broadly defined as the subjective evaluation of one’s sexual experiences, encompasses various dimensions, including sexual communication skills, the ability to express sexual needs, satisfaction with sexual activities, and emotional fulfillment (4, 5). Furthermore, sexual satisfaction has a significant association with physical and mental health, general well-being, social success, and professional achievement (5–7).

High-quality relationships marked by emotional empathy, physical attraction, love, and commitment are often accompanied by higher levels of sexual satisfaction (8). Sexual dissatisfaction can be considered as an important factor in the occurrence of marital disputes and instability of marital relationships (9–11).

### 1.1 Sexual satisfaction in Iranian couples

In Iran, marital conflicts, lack of intimacy, secret relationships, and betrayals, which may culminate in divorce, are often rooted in sexual dissatisfaction (12, 13). Cultural taboos and societal norms discourage open discussions about sexual issues, further compounding these challenges. As a result, many Iranian couples, especially those in traditional regions, remain silent on sexual matters, leading to misunderstandings and unresolved sexual problems (14–16). These issues are exacerbated by insufficient sexual education and misconceptions about sexuality. Social and cultural barriers, alongside the lack of adequate sexual health services, contribute to sexual dysfunction and dissatisfaction (15).

### 1.2 Conceptual framework

The effectiveness of sex education interventions has been confirmed by several Iranian studies (15, 17–19). Sex education typically addresses three core domains: cognition (information and knowledge), emotions (values and attitudes), and behavior (communication and sexual skills) (15, 20, 21). This study employs the Information–Motivation–Behavioral Skills (IMB) model as its theoretical framework to understand the factors influencing sexual satisfaction. The IMB model suggests that sexual health behaviors are shaped by three key components: information, motivation, and behavioral skills, all of which interact within the individual’s social environment (21).

**Figure 1:**
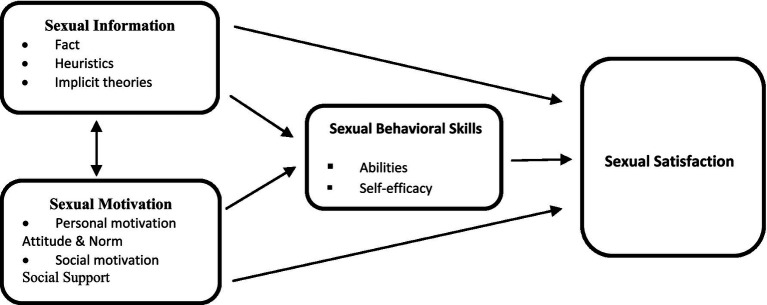
Hypothesized IMB model linking sex education program structure to sexual satisfaction.

In this context, “information” refers to factual knowledge about sexuality, which can sometimes be simplistic or misguided, leading to automatic but incorrect decisions about sexual behavior. “Motivation” includes personal attitudes and social perceptions regarding sexual health-promoting behaviors, while “behavioral skills” emphasize the human capacity to implement these behaviors effectively, including self-efficacy and communication abilities. Figure 1 illustrates this conceptual model. Based on the IMB framework, we hypothesized that an educational intervention targeting married men could enhance their sexual satisfaction by improving their knowledge, motivation, and behavioral skills. Therefore, the objective of this study was to assess the impact of an IMB-based educational intervention on the sexual satisfaction of married Iranian men.

## 2 Materials and methods

### 2.1 Study design and sampling

This quasi-experimental study was conducted among Iranian married men in Sanandaj City, Kurdistan, Iran. The sample size was calculated based on similar studies and statistical power calculations (80% power and 95% confidence interval). Each group (intervention and control) comprised 50 participants, accounting for a 10% attrition rate, with 100 individuals randomly selected from Marriage Consultant Centers. Participants were randomly assigned to groups using an interactive web response system during recruitment. The inclusion criteria included: being married, able to share experiences, of Iranian nationality, fluent in Kurdish, having been married for at least 5 years, over 18 years of age, with less than a 15-year age difference between spouses, no history of couple therapy, and at least elementary education. Participants with a history of sexual dysfunction or those unwilling to continue the study were excluded.

### 2.2 Data collection tools

The core concepts assessed were knowledge, motivation, and behavioral skills concerning sexual relationships, with sexual function as a mediator. Data were collected using three validated questionnaires: (1) a Sexual Health Questionnaire based on the Information-Motivation-Behavioral Skills (IMB) model, (2) the Male Sexual Function Questionnaire, and (3) Larson’s Sexual Satisfaction Scale.

### 2.3 Sexual health questionnaire based on (IMB) model constructs

This 51-item tool, structured into three scales and nine subscales, measures sexual health based on the IMB model. The sexual information scale (12 items) comprises three subscales: facts (7 items), exploratory discussion (2 items), and implicit theories (3 items). The sexual motivation scale (17 items) includes attitude toward sex (12 items), sexual trend (3 items), and social support (2 items). The behavioral skills scale (22 items) assesses sexual self-efficacy (11 items), behavioral skill (4 items), and behavior (7 items). The tool’s reliability and validity were confirmed by Bagheri et al. (5), with content validity indices of 0.92 and 0.90 for subscales and reliability coefficients ranging from 0.78 to 0.95.

### 2.4 The male sexual function questionnaire

This standardized questionnaire, designed to screen sexual function in men, evaluates sexual function over the past 4 weeks (22). It consists of 11 items distributed across five domains: sexual desire, erection, ejaculation, problem assessment, and overall satisfaction. Responses are scored on a 5-point Likert scale (range: 11 to 55), with higher scores indicating better sexual function. The questionnaire’s content validity was assessed by a panel of experts, and its reliability was confirmed through test–retest methods (0.93–0.97) and intraclass correlation coefficients (0.95).

### 2.5 Larson’s sexual satisfaction scale

The Persian version of Larson’s Sexual Satisfaction Scale, localized and standardized for the Iranian population by Bahrami et al. (4), was used to measure sexual satisfaction. The 25-item scale utilizes a 5-point Likert scale ranging from ‘completely agree’ (5 points) to ‘completely disagree’ (1 point), with total scores ranging from 25 to 125. Higher scores indicate greater sexual satisfaction. The scale’s reliability was determined by Cronbach’s alpha, yielding a value of 0.7.

### 2.6 Development and evaluation of the educational content

The “Buzhan” educational program, named after a Kurdish term meaning “flourishing man,” was designed based on a literature review covering individual sexual health, sexual satisfaction, sexual function, and sexual health in couples. The educational content addressed the three IMB model constructs: information, motivation, and behavioral skills. A panel of experts (including sexologists, psychologists, health professionals, and health educators) reviewed the content for accuracy, clarity, purposefulness, and simplicity.

The intervention topics included: (1) basic sexual health information (anatomy and physiology of sexual organs, common sexual problems and disorders); (2) the importance of sexual education (marital communication skills and their significance); (3) motivational factors in sexual satisfaction; (4) myths and beliefs about sexual health; (5) sexual function (sexual response cycles and common disorders); and (6) guidelines for sexual relations during pregnancy.

The educational materials were delivered digitally through booklets, stickers, podcasts, short videos, and infographics. Due to the COVID-19 pandemic, the content was shared via smartphones, incorporating multimedia messages and online discussions.

### 2.7 Procedure

#### 2.7.1 Pre-test stage

Participants in both the intervention and control groups completed a pre-test, including the three questionnaires. Given the number of questions, participants had the option to save their responses and complete the survey later.

#### 2.7.2 Training stage

The 16-week “Buzhan” program was delivered through weekly online classes and multimedia messages via smartphone in Kurdish. Online discussions, held every Wednesday for 30 min, were moderated by health professionals and psychologists using the BigBlueButton platform at Tarbiat Modares University. Multimedia messages were sent daily at 10:00 a.m., with reminders for online classes provided an hour before the session. All training was facilitated by a male sexologist. Participants received free internet access to promote adherence to the program.

#### 2.7.3 Post-test stage

Sixteen weeks after the intervention, both groups completed a post-test. The control group received the educational content after the 16-week period.

This study was approved by the Tarbiat Modares University Ethics Committee (IR.MODARES.REC.2019.038). Consent was obtained online via participants’ agreement to the survey according to the Declaration of Helsinki. Participants were briefed about the study’s purpose over the phone, and written consent was submitted electronically.

### 2.8 Statistical analysis

Statistical analysis was performed using SPSS version 16. Homogeneity between the groups was tested with Chi-square, Fisher’s exact test, and independent t-tests. Data normality was assessed using kurtosis and skewness tests. Repeated-measures ANOVA was used to analyze intergroup differences before and after the intervention. For non-normally distributed data, a nonparametric covariance test was applied.

## 3 Results

There were no significant differences between the experimental and control groups in terms of age (33.38 ± 4.43 vs. 32.73 ± 4.41 years; *p* = 0.48), spouse’s age (29.09 ± 4.81 vs. 28.76 ± 5.71 years; *p* = 0.53), or duration of marriage (3.76 ± 1.41 vs. 3.54 ± 1.54 years; *p* = 0.51). Table 1 provides a summary of the sociodemographic characteristics of the participants.

**Table 1:** Sociodemographic characteristics of the participants.

Variable	Intervention (*n* = 42)	Control (*n* = 42)	*p*-value
Education level			
High School	8 (19%)	13 (31%)	0.18*
Diploma	3 (7.1%)	6 (14.3%)	
Associate Degree	4 (9.5%)	1 (2.4%)	
Bachelor’s Degree	9 (21.4%)	15 (35.7%)	
Master’s Degree	14 (33.3%)	5 (11.9%)	
Doctorate	4 (9.5%)	2 (4.8%)	
Occupation			
Unemployed	3 (7.1%)	3 (7.1%)	0.06*
Worker	1 (2.4%)	7 (16.7%)	
Employee	28 (66.7%)	19 (45.2%)	
Student	1 (2.4%)	0 (0%)	
Free	7 (16.7%)	12 (28.6%)	
Other	2 (4.8%)	1 (2.4%)	
Spouse’s job			
Housewife	27 (64.3%)	33 (78.6%)	0.137**
Part-time Job	1 (2.4%)	3 (7.1%)	
Employee	11 (26.2%)	2 (4.8%)	
Free	1 (2.4%)	3 (7.1%)	

*Fisher’s exact test. **Chi-square test.

There was no significant difference in the mean scores for all dimensions of the IMB model and sexual satisfaction between the experimental and control groups before the intervention (*p* > 0.05). However, a significant difference was observed in all dimensions of the IMB model and sexual satisfaction between the experimental and control groups after the intervention (*p* < 0.05). Additionally, the mean scores for all dimensions of the IMB model and sexual satisfaction in the experimental group were significantly higher after the intervention (*p* < 0.05), whereas no significant changes were observed in the control group (*p* > 0.05). No significant differences were found between the groups regarding sexual function before and after the intervention (*p* > 0.05; see Table 2).

**Table 2:** Scores of the IMB model before and after the intervention.

Variable	Group	Before (Mean ± SD)	After (Mean ± SD)	*p*-value (within group)
Facts	Intervention	24.69 ± 2.15	28.66 ± 1.99	*p* < 0.001
	Control	24.92 ± 2.37	25.00 ± 2.29	0.08
Exploration	Intervention	7.66 ± 1.22	8.54 ± 0.96	*p* < 0.001
	Control	7.88 ± 1.38	7.90 ± 1.33	0.31
Implicit theories	Intervention	8.42 ± 1.65	11.16 ± 1.84	*p* < 0.001
	Control	8.61 ± 1.62	8.66 ± 1.60	0.15
Personal motivation	Intervention	46.73 ± 3.47	49.42 ± 3.28	*p* < 0.001
	Control	47.04 ± 3.65	47.11 ± 3.68	0.08
Social support	Intervention	3.19 ± 0.80	4.19 ± 0.91	*p* < 0.001
	Control	3.52 ± 1.13	3.57 ± 1.12	0.41
Sexual function	Intervention	41.26 ± 5.56	41.28 ± 5.15	*p* = 0.09
	Control	41.05 ± 6.09	41.23 ± 6.07	*p* = 0.10
Sexual satisfaction	Intervention	73.83 ± 5.06	84.35 ± 4.43	*p* < 0.001
	Control	74.04 ± 6.23	74.26 ± 5.72	*p* = 0.53

The educational intervention was effective in improving sexual satisfaction, as indicated by the significant difference in post-intervention scores (*p* = 0.001). In the experimental group, 60% of the variation in sexual satisfaction post-intervention could be attributed to the educational intervention (Table 3). The intervention group’s significant improvement in sexual satisfaction confirms the effectiveness of the educational program.

**Table 3:** Covariance analysis of sexual satisfaction scores.

Source	Total squares	Df	Mean squares	F	*p*-value	Effect size	Test power
Pre-test	718.23	1	718.23	40.64	*p* = 0.53	0.33	1.0
Group	2187.01	1	2187.01	123.74	*p* < 0.001	0.60	1.0
Error	1431.52	81	0.82				

## 4 Discussion

This study aimed to evaluate the effectiveness of sexual satisfaction training programs using the IMB model in married men in Sanandaj, Iran. The findings demonstrate that the intervention significantly impacted all model constructs and sexual satisfaction, except for sexual function.

The educational intervention program in this study effectively increased participants’ sexual information. Specifically, 60, 31, and 40% of the mean differences in facts, exploration, and implicit theories in the control group were attributed to the educational intervention. This is consistent with Vural and Temel’s and Suwarni et al. study, which found that educational programs based on the IMB model improved participants’ sexual knowledge (7, 23). Sexual education can address ambiguous beliefs about sexuality, enhancing access to accurate information (22).

The intervention significantly enhanced sexual motivation, correcting traditional attitudes that hinder sexual satisfaction (e.g., performing duty, obedience, shame, dominance in the relationship, requesting a relationship by the spouse, violence and power-seeking behaviors), to experience satisfying sex with their spouse. The possible explanation is although sexual orientations are inherent and involuntary, sexual attitudes and behaviors are learnable. These findings align with Mallory et al. (24) and others, suggesting that informed sexual attitudes foster better sexual expression and experiences (25, 26). By addressing the modification of harmful beliefs and attitudes, the program helped men experience more fulfilling sexual relationships with their spouses.

Social support emerged as a critical factor in improving sexual satisfaction. Participants who received social support reported higher marital satisfaction, consistent with other studies (27, 28). This highlights the importance of active social and community support systems, especially in cultures where formal sexuality education services are lacking or stigmatized (15).

The intervention also positively influenced sexual skills, self-efficacy, and behavior. Participation in the sexual training program led to reduced incoherence and conflict in sexual relations, and increased couples’ sexual knowledge and skills. The perception of sexual self-efficiency is associated with the ability of individuals to perform sexual behaviors correctly and satisfactorily (15). While the experimental group demonstrated significant improvements in these areas, the results for sexual function (desire, ejaculation, erection, and satisfaction) remained largely unchanged, with minimal improvement. This discrepancy may be due to the nature of the online/offline intervention during the COVID-19 pandemic and the limited follow-up period, which might not have been sufficient to observe changes in sexual function.

The increase in sexual satisfaction in the experimental group aligns with prior research (29, 30), confirming that educational interventions can significantly improve sexual satisfaction. From the perspective of social exchange theory, marital satisfaction is linked to sexual satisfaction and vice versa (31). In this context, improving positive sexual behaviors while reducing negative ones enhances both sexual and marital satisfaction (32).

### 4.1 Limitations

The main limitation of this study was the in-person educational intervention sessions due to the COVID-19 pandemic. The inability to include women in the study was another limitation, as was the cultural sensitivity surrounding sexual discussions in Iran. This focus on male sexual satisfaction may have led to sensitivity bias in some participants, which should not be interpreted as an endorsement of male dominance in sexual relationships, but rather as a practical decision based on cultural constraints.

## 5 Conclusion

The educational program effectively improved sexual satisfaction and related constructs (information, motivation, and behavioral skills) in married men, except for sexual function. The program addressed critical gaps in sexual information, attitudes, and self-efficacy, empowering participants to improve their sexual well-being. Despite cultural barriers, the program succeeded in enhancing sexual knowledge and satisfaction, suggesting its potential for broader application of the IMB model.

Future interventions could consider simultaneously involving women and men (couples) with a focus on improving sexual function in longitudinal studies with longer follow-up periods that are more appropriate for assessing sustained effects.

## Data Availability

The original contributions presented in the study are included in the article/supplementary material, further inquiries can be directed to the corresponding author.
